# A Large Congenital Anterior Urethral Diverticulum in a 14-Month-Old Boy

**DOI:** 10.7759/cureus.18104

**Published:** 2021-09-19

**Authors:** Ali Alyami, Ahmed AlShammari, Tariq Burki

**Affiliations:** 1 Urology Division, Department of Surgery, King Abdulaziz Medical City, Ministry of National Guard Health Affairs, Riyadh, SAU; 2 Department of Pediatric Urology, King Abdullah Specialized Children Hospital, King Abdulaziz Medical City, Ministry of National Guard Health Affairs, Riyadh, SAU

**Keywords:** urethrocystoscopy, open diverticulectomy, urethral obstruction in children, anterior urethral valve, congenital anterior urethral diverticulum

## Abstract

Congenital anterior urethral diverticulum is a rare condition causing lower urinary tract obstruction in children. It usually arises from the ventral aspect of the anterior urethra, mostly located at the penoscrotal junction. We report a case of a 14-month-old baby boy who presented with a soft ventral swelling over the distal penile urethra, difficulty in passing urine, and a history of recurrent febrile urinary tract infections. A retrograde urethrogram revealed a large distal anterior urethral diverticulum. He underwent diverticulectomy and primary repair with no post-operative complications. The treatment of these depends on the size of the diverticulum and the degree of obstruction. In cases of a large anterior urethral diverticulum, open diverticulectomy and primary repair are recommended.

## Introduction

Urethral diverticulum is defined as epithelialized, saccular dilatation that is separate from the urethra but communicates with the lumen through a discrete orifice [1]. Congenital anterior urethral diverticulum (CAUD) is an extremely rare entity in children and is defined as an outpouching of the anterior urethra through the corpus spongiosum [2]. The exact number of cases reported so far in children is not known. Paulhac et al. reported up to 260 cases in 2003 but with no clear distinction between anterior urethral valve (AUV) and CAUD [3].

Clinical presentation varies depending on the age and severity of urinary obstruction. It includes a poor urinary stream, post-void dribbling, recurrent urinary tract infections (UTI), and ventral penile swelling [2]. Detailed clinic examination and imaging studies, such as retrograde urethrogram (RUG) and micturating cystourethrogram (MCUG), help in diagnosing the condition [4-5]. The treatment options include either watchful waiting in minor cases, or endoscopic, or open surgical excision depending on the symptoms, type and size of the diverticulum, and upper urinary tract changes [4]. In this report, we describe the presentation, diagnosis, and treatment of a large anterior urethra diverticulum in a 14-month-old boy with a brief review of the literature.

## Case presentation

**Figure 1:**
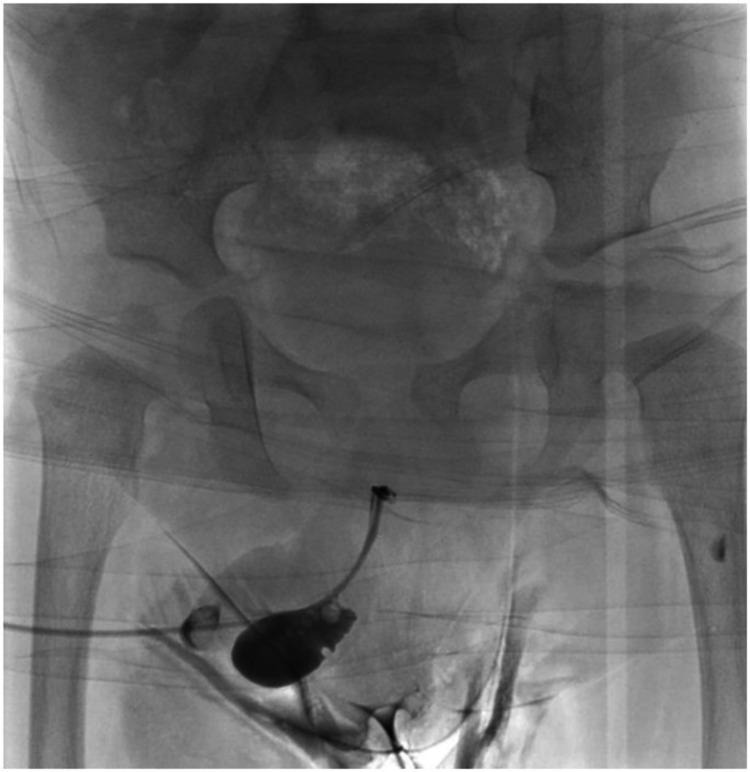
Retrograde urethrogram shows outpouching along the ventral aspect of the anterior urethra

A 14-month-old baby boy was referred to our pediatric urology clinic from a secondary care center with a history of two febrile UTIs and ballooning of the penis during micturition requiring manual compression by his mother for complete evacuation. The referring team was suspecting megalourethra. Physical examination of the genitalia revealed an uncircumcised penis, soft swelling over the mid-shaft area ventrally, and normal testes in the scrotum. A RUG from referring hospital showed an outpouching along the ventral aspect of the distal urethra (Figure 1).

**Figure 2:**
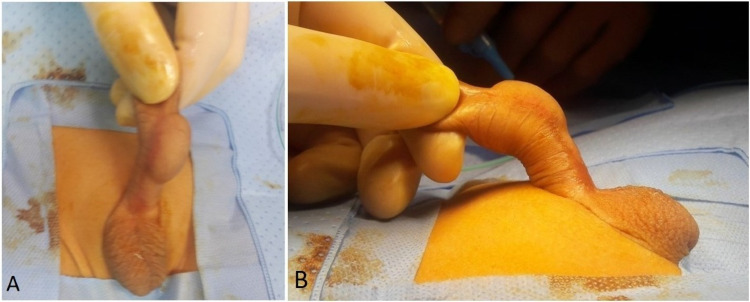
The examination under anesthesia Picture A shows the front view, while picture B shows the side view of the diverticulum.

Cystogram could not be done due to difficulty in the insertion of the catheter into the bladder, perhaps an uncooperative child. An ultrasound scan of the swelling showed a well-defined anechoic cystic structure seen in soft tissues of the penis with normal renal tract. He underwent examination under anesthesia, which showed a very patulous distorted shaft skin over the ventral aspect (Figures 2A-2B).

**Figure 3:**
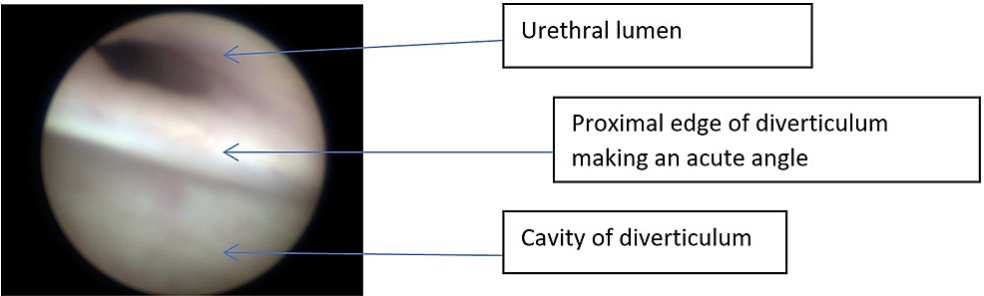
The cystourethroscopy shows the edge of the anterior urethral diverticulum.

**Figure 4:**
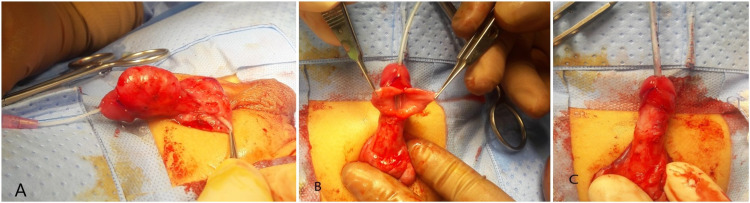
A. After complete degloving of the penis and injection of saline into the diverticulum with an 8F NG tube, the extent and severity of the diverticulum is displayed. B. The diverticulum opened up in the midline. It was excised flush with the urethra followed by urethroplasty. C. Final appearance after applying a corpus spongiosum flap to cover the repair.

Cysto-urethroscopy showed normal bladder, ureteric orifices, and a posterior urethra with no evidence of the posterior urethral valves (PUV). The anterior urethra revealed a large distal urethral diverticulum with an acute edge proximally with no evidence of anterior urethral valves (AUV) (Figure 3). He had complete degloving of the penis and dissection of the diverticulum. The corpus spongiosum was deficient on the left side of the midline along the entire length of the diverticulum. The diverticulum was opened in the midline, and the excess tissues were excised flush with the edges of the normal urethra over an 8F NG tube. The edges were stitched back with no tension using 6-0 PDS (Figures 4A-4C). Circumcision was performed at the end of the procedure. The catheter was removed after a week without any complications. He was seen in the clinic after six months with no issues reported by the parents and had normal examination findings.

## Discussion

CAUD usually presents in the first few months of life but may present at any age [6]. The etiology of this condition remains unclear. It has been suggested that the lack of a corpus spongiosum results in urethral dilatation leading to a diverticulum [2]. As we found in the current case the corpus spongiosum was deficient on one side. Another theory suggests that during embryogenesis, the diverticulum of the urethra develops because of epidermal pockets communicating with the ventral urethral wall. As the anterior urethral tube forms, the urethral groove may form a congenital cyst and the diverticulum could be formed as a result of the spontaneous rupture of the cyst into the urethral lumen [2]. In the majority of the cases, it is located at the penoscrotal junction while in one-third of the cases it occurs distally [7].

Some children may present antenatally with an obstructive picture on antenatal ultrasound but the majority present postnatally with lower urinary tract symptoms including difficulty in micturition, dribbling of urine, poor urinary stream, and/or UTI. In severe cases, they may present with renal failure and gross upper tract changes [6-8]. Parents usually report that the child has never had a good urinary stream since birth. They have noticed a cystic swelling in the penile urethra, which increases in the size during micturition, and on compression, urine is seen dribbling out of the external meatus, with a reduction in the size of the swelling [7]. Such swelling can be confused with scaphoid megalourethra, which is much more severe in appearance, and corporal deficiency can be appreciated on clinical examination [1]. As there is some degree of deficiency of corpus spongiosum in CAUD so it can be regarded as a minor spectrum of megalourethra.

**Figure 5:**
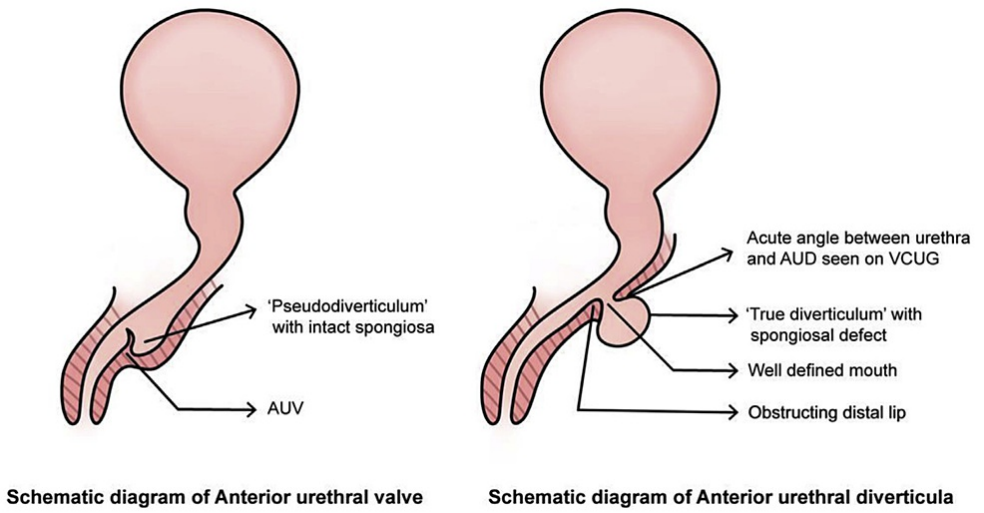
Schematic diagram shows the differences between two conditions Printed with permission from Jain et al. [8].

The other main differential includes AUV, which is a mucosal fold in the distal urethra similar to the PUV [8]. In some cases, there is significant proximal dilatation, which leads to confusion in differentiating it from CAUD. Many authors believe that CAUD and AUV are the same entity but with a different spectrum of severity [9-10]. Others believe these to be totally different entities [10]. One reason for this confusion is that in both conditions, there is dilatation of the distal urethra, which may appear similar. The other reason is that in CAUD, there is an acute angulation at the proximal end of the diverticulum, which during micturition not only causes filling of the diverticulum but also lifts it up and causes obstruction of the urethra leading to obstructive symptoms similar to AUV [8]. Jain et al. have suggested a way to differentiate between AUV and CAUD. In AUV, the proximal lip of the diverticulum is obtuse while in CAUD, it is acute (Figure 5) [8]. This may be evident on urethrogram, which can help in differentiating the two conditions or during cystoscopy like our case, as can be seen in Figure 3. Investigations such as RUG and MCUG can help in the diagnosis of the condition. Additionally, these may also give important information about bladder appearance and the presence of vesicoureteral reflux [4-5].

If the condition is minor, it can be either left as such or treated cystoscopically by incising the distal lip of the diverticulum [8-11]. This will stop the distal mucosal fold from acting as an obstructive valve and the diverticulum will become a self-emptying diverticulum. On the other hand, if the diverticulum is big, open surgical diverticulectomy is required making sure that the distal obstructive lip is dealt with properly; otherwise, the obstructive symptoms may persist postoperatively [12].

## Conclusions

CAUD is a rare entity causing lower urinary tract obstruction in the pediatric age group. Utilizing imaging studies, such as RUG and MCUG, can help in the diagnosis of the condition. The treatment options are varied from watchful waiting to open surgical excision, in the case of the large anterior urethral diverticulum, open diverticulectomy and primary repair are recommended.
